# Antihypertensive effects of inducible nitric oxide synthase inhibition in experimental pre-eclampsia

**DOI:** 10.1111/jcmm.12106

**Published:** 2013-07-24

**Authors:** Lorena M Amaral, Lucas C Pinheiro, Danielle A Guimaraes, Ana CT Palei, Jonas T Sertório, Rafael L Portella, Jose E Tanus-Santos

**Affiliations:** a Department of Pharmacology, Faculty of Medicine of Ribeirao Preto, University of Sao PauloRibeirao Preto, SP, Brazil; b Department of Pharmacology, Faculty of Medical Sciences, State University of CampinasCampinas, SP, Brazil

**Keywords:** Pre-eclampsia, inducible nitric oxide synthase, nitrotyrosine, oxidative stress, reduced uteroplacental perfusion pressure, RUPP

## Abstract

Upregulation of inducible nitric oxide synthase (iNOS) has been reported in both experimental and clinical hypertension. However, although pro-inflammatory cytokines that up-regulate iNOS contribute to pre-eclampsia, no previous study has tested the hypothesis that a selective iNOS inhibitor (1400 W) could exert antihypertensive effects associated with decreased iNOS expression and nitrosative stress in pre-eclampsia. This study examined the effects of 1400 W in the reduced uteroplacental perfusion pressure (RUPP) placental ischaemia animal model and in normal pregnant rats. Sham-operated and RUPP rats were treated with daily vehicle or 1 mg/kg/day N-[3-(Aminomethyl) benzyl] acetamidine (1400 W) subcutaneously for 5 days. Plasma 8-isoprostane levels, aortic reactive oxygen species (ROS) levels and nicotinamide adenine dinucleotide phosphate (NADPH)-dependent ROS production were evaluated by ELISA, dihydroethidium fluorescence microscopy and lucigenin chemiluminescence respectively. Inducible nitric oxide synthase expression was assessed by western blotting analysis and aortic nitrotyrosine was evaluated by immunohistochemistry. Mean arterial blood pressure increased by ∼30 mmHg in RUPP rats, and 1400 W attenuated this increase by ∼50% (*P* < 0.05). While RUPP increased plasma 8-isoprostane levels, aortic ROS levels, and NADPH-dependent ROS production (*P* < 0.05), treatment with 1400 W blunted these alterations (*P* < 0.05). Moreover, while RUPP increased iNOS expression and aortic nitrotyrosine levels (*P* < 0.05), treatment with 1400 W blunted these alterations (*P* < 0.05). These results clearly implicate iNOS in the hypertension associated with RUPP. Our findings may suggest that iNOS inhibitors could be clinically useful in the therapy of pre-eclampsia, especially in particular groups of patients genetically more prone to express higher levels of iNOS. This issue deserves further confirmation.

## Introduction

Pre-eclampsia is an important complication of pregnancy and multiple hypotheses have been proposed to elucidate its pathogenesis, including inadequate placentation that activates complex mechanisms leading to systemic maternal vascular dysfunction [1–5]. Indeed, while cytotrophoblast migrate towards the uterine spiral arterioles and undergo differentiation into endothelial cells during normal pregnancy, the invasion of spiral arteries is limited in pre-eclampsia, thus reducing uteroplacental perfusion and causing ischaemia of the placenta [6, 7].

Nitric oxide plays an important role in cardiovascular homoeostasis, and it is formed from L-arginine by a family of nitric oxide synthases, including inducible nitric oxide synthase (iNOS). While nitric oxide concentrations are critically regulated, abnormally high nitric oxide concentrations may be deleterious to the vessels, especially when nitric oxide reacts with other reactive oxygen species (ROS) such as superoxide to form peroxynitrite [8]. In fact, increased oxidative stress has been reported in pre-eclampsia, and this alteration contributes to the pathogenesis of this critical condition [8]. However, although elevated levels of circulating pro-inflammatory cytokines that up-regulate iNOS have been shown in pre-eclampsia [9–11], no previous study has examined whether up-regulated iNOS expression contributes to the vascular alterations found in this condition.

In this study, we tested the hypothesis that upregulation of vascular iNOS expression may promote vascular oxidative and nitrosative stress and hypertension in pregnant rats with placental ischaemia (reduced uteroplacental perfusion pressure-RUPP animal model) [12, 13]. In addition, it has been suggested by us that selective iNOS inhibition could exert antihypertensive effects in this experimental model. These hypotheses are supported by previous results implicating iNOS in experimental hypertension [14]. In contrast with our hypothesis, there is evidence that iNOS has an important role in the renal hemodynamic changes of pregnancy, and iNOS inhibition decreased glomerular filtration in pregnant rats [15], although renal iNOS apparently is not up-regulated in RUPP rats [12].

## Materials and methods

### Animals and treatment

This study complied with guidelines of the Faculty of Medicine of Ribeirao Preto, University of Sao Paulo, and the animals were handled according to the guiding principles published in the National Institutes of Health Guide for the Care and Use of Laboratory Animals. Female Wistar rats obtained from the colony at the University of Sao Paulo (Ribeirao Preto Campus, Brazil) were used in this study. Rats were maintained on a 12-hr light/dark cycle at room temperature (22–25°C) with free access to standard rat chow and water. On day 14 of gestation, rat dams were randomly assigned to either RUPP or Sham pregnant control groups. The RUPP procedure is a well-established model of pre-eclampsia and has been previously described in detail [12, 13]. All surgical procedures were carried out under 2% isoflurane anaesthesia. Briefly, pregnant rats in the RUPP group underwent a midline incision and the lower abdominal aorta was isolated. A silver clip (0.203 mm) was placed around the aorta above the iliac bifurcation. To prevent augmentation of blood flow to the uterus *via* the ovarian arteries, silver clips (0.100 mm) were also placed on the branches of both ovarian arteries that supply the uterus. Sham procedure consisted of abdominal incision, isolation of the abdominal aorta and ovarian arteries. When the clipping procedure resulted in total reabsorption of the foetuses, the rats were excluded from the study.

Sham-operated and RUPP rats were treated with daily vehicle or 1 mg/kg/day N-[3-(Aminomethyl) benzyl] acetamidine (1400 W) subcutaneously for 5 days. This drug is a highly selective iNOS inhibitor with irreversible or extremely slowly reversible effects, and at least 1000-fold more potent selectivity for iNOS than for endothelial NOS in rat aortic rings [16].

### Measurement of arterial blood pressure

On day 18 of gestation, the rats (*n* = 10–12/group) were anaesthetized again and implanted with an indwelling polyethylene catheter (PE50) inserted into the left carotid that was tunnelled under the skin and externalized at the back of the neck. On the following day (day 19 of gestation), the rats were placed in individual restraining cages and acclimatized. The mean arterial pressure (MAP) was recorded in conscious rats for 1 hr using a data acquisition system (MP150CE; Biopac Systems Inc., Goleta, CA, USA) connected to a computer (Acknowledge 3.2, for Windows). Then the rats were anaesthetized again, killed and blood and tissue samples were collected to carry out all the analyses described below.

### Measurement of plasma 8-isoprostane concentrations

To evaluate oxidative stress, plasma 8-isoprostane (8-isoPGF_2α_) concentrations (*n* = 5–7/group) were measured in duplicate with commercially available ELISA kits (Cayman Chemical Company, Ann Arbor, MI, USA), according to manufacturer's instructions.

### Assessment of vascular ROS production

Dihydroethidium (DHE) was used to evaluate aortic *in situ* ROS production by fluorescence microscopy as described previously [17]. Aortic cryosections (5-μm thick; five sections/animal; *n* = 6–8 animals/group) were incubated at 37°C with DHE (1 μmol/l) for 30 min. After triple washing with phosphate buffer, the sections were examined by fluorescence microscopy (Leica Imaging Systems Ltd., Cambridge, UK) and the image was captured at ×400. Red fluorescence from 20 fields around the vessel were evaluated using ImageJ software (http://rsbweb.nih.gov/ij/), and the arithmetic mean of the fluorescence from the 20 fields was calculated for each slide, as described before [18].

### Measurement of NADPH-dependent ROS production

NADPH-dependent ROS production was measured in aortic rings from all experimental groups (*n* = 6–8/group). Aortic rings were transferred to luminescence vials containing 1 ml of Hanks' buffer, pH 7.2. After equilibration and background counts, a non-redox-cycling concentration of lucigenin (5 μmol/l) and β-NADPH (12 μmol) was automatically added and the luminescence counts were measured continuously for 15 min. in a Berthold FB12 single-tube luminometer at 37°C. Background signals from the aortic rings were subtracted from the β-NADPH-driven signals and the results were normalized for the dry weight and reported as lucigenin chemiluminescence/mg of dry tissue, as described previously [19].

### Western blotting analysis of iNOS

Vascular iNOS expression was evaluated in the aortas (*n* = 5–7/group). Briefly, aortic extracts were homogenized on cold RIPA-buffer. One hundred micrograms of protein extracts was separated by SDS-PAGE using an 8% polyacrylamide gel. The proteins were transferred onto nitrocellulose membranes (GE Healthcare, Madison, WI, USA). After blocking in 5% milk, membranes were incubated overnight at 4°C with primary antibody directed against iNOS (1:1000; Millipore, Billerica, MA, USA). Then the membranes were incubated with horseradish peroxidase (HRP)-secondary goat anti-rabbit antibody (1:2000; Millipore) and revealed with ECL chemiluminescence kit (GE Healthcare). Inducible nitric oxide synthase expression was normalized with respect to β-actin expression (1:1000; Millipore).

### Immunohistochemistry to detect vascular nitrotyrosine

To measure vascular nitrotyrosine levels in the aortic media layer, thoracic aortas were fixed in 4% phosphate-buffered paraformaldehyde, pH 7.4, and embedded in paraffin blocks. Sections (5 μm thick; *n* = 8–10/group) were incubated with 3% H_2_O_2_ in water for 5 min. to block endogenous peroxidase activity, and then incubated overnight at 4°C with the primary antibody anti-nitrotyrosine (1:50; Millipore) and the secondary antibody (1:200; Millipore) was added to the sections for 1 hr at room temperature, as previously described [20]. Antigen was visualized with a labelled streptavidin biotin peroxidase technique (Vectastain ABC kit; Vector Laboratories Inc., Burlingame, CA, USA) with diaminobenzidine (DAB) substrate. Sections were then counterstained with haematoxylin, and examined using light microscopy (Leica Imaging Systems Ltd.) and the image was captured at ×400. The binding is shown as a dark brown colour. Immunoreactivity intensity was measured using the ImageJ Program (National Institutes of Health). The assessment of nitrotyrosine by immunohistochemistry was made by quantifying immunoreactivity intensity from 20 fields randomly selected around the media vessel circumference. The arithmetic mean of the immunoreactivity intensity from the 20 fields was calculated for each slide (*n* = 8–10). The number of fields per slide corresponds to ∼20–30% of the total aortic area being studied and has led to interassay coefficients of variations of less than 4%. Omission of the primary antibody was used to determine the background generated during the detection assay. Observers were blinded to the identity of the treatment groups.

### Statistical analysis

The results are expressed as means ± SEM. Comparisons between groups were assessed by two-way anova. A probability value *P* < 0.05 was considered significant.

## Results

### iNOS inhibitor attenuates hypertension associated with RUPP

**Fig. 1:**
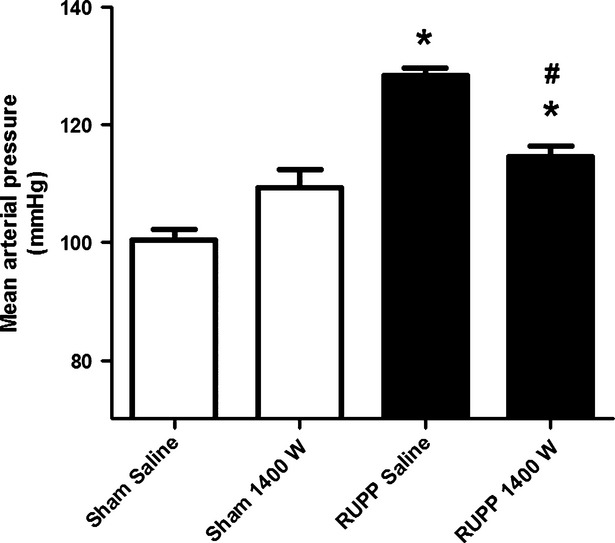
Treatment with 1400 W attenuates the reduced uterine perfusion pressure (RUPP)-induced arterial hypertension. Data are shown as mean ± SEM (*n* = 10–12/group); *P < 0.05 *versus* Sham Saline group; ^#^*P* < 0.05 *versu*s RUPP Saline group.

To examine whether iNOS plays a role in RUPP-induced hypertension, we administered the iNOS inhibitor 1400 W to rats. Figure 1 clearly shows increased MAP in RUPP rats compared with the Sham Saline group (MAP = 128 ± 1 *versus* 100 ± 2 mmHg; *P* = 0.001; Fig. 1). Treatment with 1400 W attenuated this increase in MAP by ∼50% (MAP = 114 ± 2.0 in the RUPP 1400 W group; *P* = 0.001; Fig. 1).

### iNOS inhibitor attenuates systemic and vascular oxidative stress associated with RUPP

**Fig. 2:**
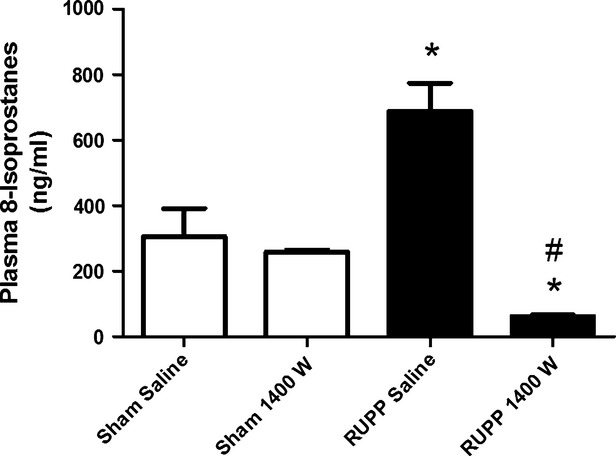
Treatment with 1400 W blunts reduced uterine perfusion pressure (RUPP)-induced increases in plasma 8-isoprostane (ng/ml) concentrations. Data are shown as means ± SEM (*n* = 5–7/group); *P < 0.01 *versus* Sham Saline group; ^#^*P* < 0.01 *versu*s RUPP Saline group.

**Fig. 3:**
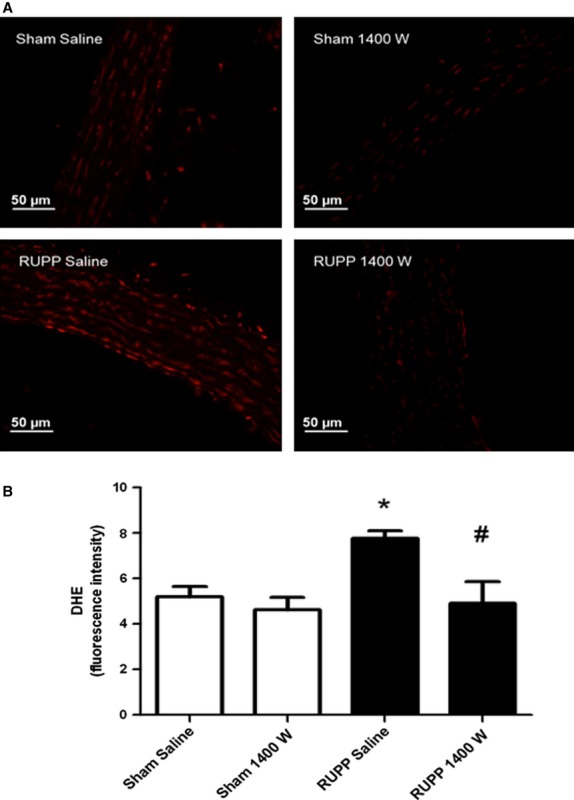
Treatment with 1400 W blunts reduced uterine perfusion pressure (RUPP)-induced increases in aortic reactive oxygen species levels measured by dihydroethidium (DHE) fluorescence. (**A**) Representative photomicrographs (original magnification ×400) of arteries incubated in the presence of DHE, which produces a red fluorescence when oxidized to hydroxyethidium by O_2_^−^. (**B**) Fluorescence intensity in each experimental group. Data are shown as means ± SEM (*n* = 6–8/group); *P < 0.05 *versus* Sham Saline group; ^#^*P* < 0.05 *versu*s RUPP Saline group.

**Fig. 4:**
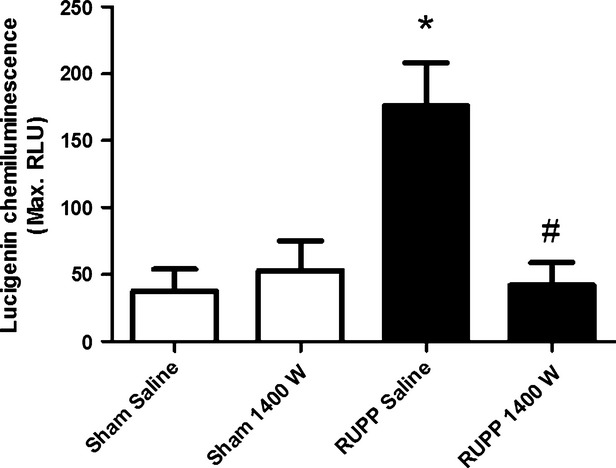
Treatment with 1400 W blunts reduced uterine perfusion pressure (RUPP)-induced increases in aortic NADPH-dependent reactive oxygen species production measured by lucigenin chemiluminescence. Data are shown as mean ± SEM (*n* = 6–8/group); *P < 0.05 *versus* Sham Saline group; ^#^*P* < 0.05 *versu*s RUPP Saline group.

Figure 2 shows increased circulating 8-isoprostane levels (689 ± 85 *versus* 305 ± 85 ng/ml; *P* = 0.001) and Figure 3 shows increased aortic ROS levels associated with RUPP [7.7 ± 0.3 *versus* 5.2 ± 0.4 arbitrary units (A.U.); *P* = 0.001]. Interestingly, the iNOS inhibitor 1400 W completely blunted these alterations (Figs 2 and 3; both *P* < 0.05). To further confirm these results showing less oxidative stress associated with 1400 W, NADPH-dependent ROS production was measured in aortic rings from rats. While RUPP was associated with increased lucigenin chemiluminescence signals, consistent with increased aortic NADPH oxidase activity in the RUPP Saline group compared with the Sham Saline group, treatment with 1400 W completely blunted this alteration (176 ± 32 *versus* 42 ± 17 A.U.; *P* = 0.018, Fig. 4).

### iNOS inhibitor blunts the increases in iNOS expression associated with RUPP

**Fig. 5:**
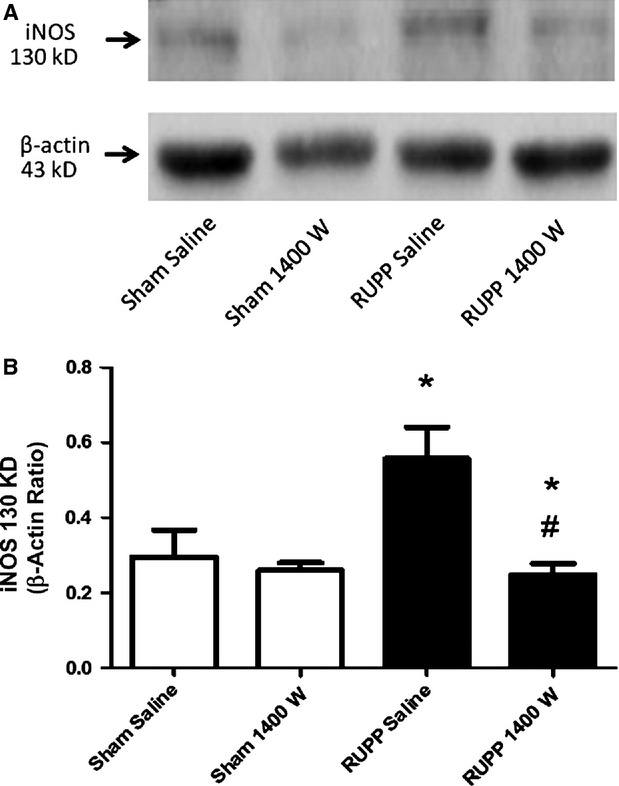
Treatment with 1400 W blunts reduced uterine perfusion pressure (RUPP)-induced increases in aortic inducible nitric oxide synthase (iNOS) expression. (**A**) Representative Western blot gel showing iNOS expression in the aortas from rats. (**B**) Bar graph showing the densitometric data. β-Actin content was used for normalization. Data are shown as means ± SEM (*n* = 5–7/group); *P < 0.05 *versus* Sham Saline group; ^#^*P* < 0.05 *versu*s RUPP Saline group.

While RUPP was associated with increased aortic iNOS expression, treatment with 1400 W blunted this alteration in the RUPP 1400 W group (0.55 ± 0.08 *versus* 0.25 ± 0.03; *P* = 0.011; Fig. 5).

### iNOS inhibitor attenuates the increases in vascular nitrosative stress associated with RUPP

**Fig. 6:**
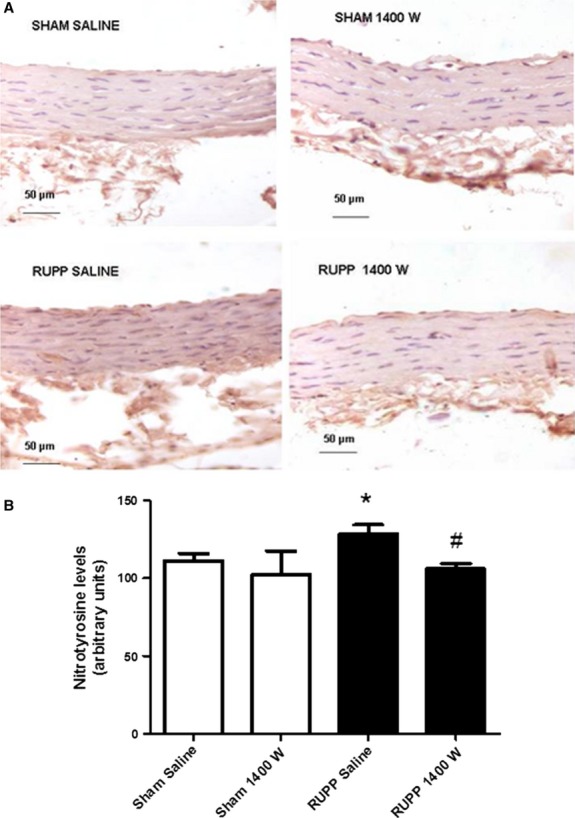
Treatment with 1400 W blunts reduced uterine perfusion pressure (RUPP)-induced increases in aortic nitrotyrosine levels. (**A**) Representative immunohistochemistry photomicrographs showing nitrotytirosine levels in aortas from all experimental groups (×400). (**B**) Bar graph shows the quantification of brown staining. Data are shown as means ± SEM (*n* = 8–10/group); *P < 0.05 *versus* Sham Saline group; ^#^*P* < 0.05 *versu*s RUPP Saline group.

Figure 6 shows that RUPP was associated with increased vascular nitrotyrosine levels (129 ± 6 *versus* 111 ± 5 A.U.; *P* = 0.013). Treatment with 1400 W attenuated this alteration, as revealed by lower aortic nitrotyrosine levels in the RUPP 1400 W group (106 ± 3 A.U.) compared with the RUPP Saline group (Fig. 6; *P* = 0.012).

## Discussion

This is the first study to show that treatment with iNOS inhibitor 1400 W exerts antihypertensive effects in the RUPP model of pre-eclampsia. Indeed, the most important findings of this study were as follows: (*i* ) vascular iNOS expression is up-regulated in the RUPP model of pre-eclampsia; (*ii* ) the iNOS inhibitor 1400 W blunted the increases in iNOS expression as well as the oxidative and nitrosative stress associated with RUPP; (*iii* ) the iNOS inhibitor 1400 W decreased MAP in the RUPP model. Together, these results suggest a significant contribution of iNOS to promote oxidative and nitrosative stress and hypertension in this animal model. Our findings may have important clinical implications because they suggest that iNOS inhibition may be a relevant strategy in the management of this critical condition.

The RUPP animal model has been widely accepted as an interesting animal model to reproduce many features of clinical pre-eclampsia [12, 13, 21–25]. Previous studies have shown that placental ischaemia found in the RUPP model promotes oxidative stress and contributes to hypertension [22]. In fact, RUPP increased placental levels of 8-isoprostane (a marker of oxidative stress) and treatment with the antioxidant tempol attenuated the hypertension in the RUPP model [22]. In line with this previous study, we found increased circulating levels of 8-isoprostane and increased vascular ROS levels, which were also associated with increased vascular NADPH-dependent ROS production in RUPP rats. NADPH oxidase is a major source of superoxide in the vasculature [26–28], and therefore the increased NADPH oxidase activity reported here in RUPP rats is consistent with an important contribution of oxidative stress to hypertension in this animal model. Together, these findings suggest that placental ischaemia promotes mechanisms increasing oxidative stress that are not limited to the placenta [22] and directly involve the vasculature as well.

While previous studies have implicated pro-inflammatory cytokines that are known to up-regulate iNOS in pre-eclampsia [9–11], this is the first study to show increased iNOS expression in the vasculature of RUPP rats. Interestingly, a previous study consistently showed increased levels of the inflammatory cytokine TNF-α in the RUPP model, and the infusion of this cytokine into pregnant rats increased blood pressure [13]. However, although TNF-α increases iNOS expression in different cell types [29], the effects of iNOS inhibitors have never been examined in pre-eclampsia models. Our results show that treatment with 1400 W exerted relevant antihypertensive effects that were associated with blunted iNOS expression upregulation. Whereas 1400 W is a highly selective iNOS inhibitor [16], it may also decrease iNOS expression, as reported here and in other studies [30–32]. This important effect of 1400 W may explain the attenuation of RUPP-induced increases in vascular nitrotyrosine levels, which may reflect vascular peroxinitrite formation. Consistent with this suggestion, it is well known that increased ROS levels react with increased amounts of nitric oxide produced by iNOS to form peroxynitrite, a powerful oxidizing agent that may contribute to the pathogenesis of many cardiovascular diseases [33, 34], including pre-eclampsia [8, 10]. While our findings do not show a precise mechanism for the antihypertensive effects of 1400 W, it is highly probable that this drug attenuated RUPP hypertension by decreasing oxidative and nitrosative stress. Indeed, conduit vessels have a relative role in long-term regulation of arterial pressure. However, it is reasonable to expect that the vascular alterations reported here may well be found in small, resistance vessels, which are more relevant to control arterial pressure. Moreover, iNOS upregulation impairs endothelial NOS (eNOS)-derived nitric oxide formation causing endothelial dysfunction [35] and also increases arginase activity decreasing nitric oxide bioavailability [36]. All these mechanisms may have been inhibited by 1400 W, and may explain its antihypertensive effects reported here.

We used the highly selective iNOS inhibitor 1400 W in this study. Although this drug directly inhibits iNOS [16], other studies found that 1400 W decreases iNOS expression [30, 31]. This iNOS inhibitor was considered to have absolute specificity to inhibit iNOS as compared to eNOS and this effect was a time-dependent result of 1400 W interactions with iNOS that develop slowly [16]. Although we have not measured iNOS activity in this study, the 1400 W that we used (1 mg/kg) totally prevented vascular injury attributable to LPS-induced iNOS in rats (ED_50_ = 0.3 mg/kg) [16]. Interestingly, other iNOS inhibitors were shown to increase blood pressure in normal rats [15], possibly as a result of non-selective iNOS inhibition, whereas our findings showed antihypertensive effects with 1400 W, possibly as a result of selective iNOS inhibition.

Although no previous study has examined the effects of iNOS inhibitors in pre-eclampsia models, iNOS upregulation has been implicated in both animal models and clinical hypertension [14, 35, 37]. In parallel with our findings, aminoguanidine (an iNOS inhibitor) decreased iNOS expression and lowered nitrotyrosine and superoxide levels in spontaneously hypertensive rats in association with suppression of hypertension development [14]. Moreover, iNOS inhibition decreased vascular nitrotyrosine levels associated with ageing and improved endothelial function, probably as a result of attenuated peroxynitrite formation [32].

In contrast with the antihypertensive effects that we found with 1400 W, a previous study examining the effects of other iNOS inhibitors showed that acute iNOS inhibition increased arterial pressure in normal pregnant rats [15]. The authors have examined the effects of iNOS inhibitors only in normal rats, and not in the RUPP model, and they showed that iNOS inhibition attenuated the increase in glomerular filtration rate observed in pregnant rats [15], thus suggesting that iNOS has a role in mediating the renal hemodynamic changes during pregnancy. Moreover, the same group has shown that renal iNOS expression decreases by 11% in the RUPP model as compared with normal pregnancy [12]. Therefore, on the basis of this information, we believe that the antihypertensive effects of 1400 W in the RUPP model do not necessarily involve alterations in renal hemodynamics. However, this suggestion remains to be examined. It is also possible that differences between studies may reflect differences between tissues being studied (renal *versus* aortic) or differences between particular iNOS inhibitors used in the studies. It should also be noted that previous studies were carried out in Sprague-Dawley rats [12, 15], whereas we have used Wistar rats.

Although vascular iNOS expression doubled in RUPP animals compared with the Sham group, other factors may contribute to hypertension in this model, as discussed above. For example, increased oxidative stress uncouples endothelial NOS and promotes vascular dysfunction [33]. This alteration may reflect potential contrasting roles of different NOS isoforms in normal pregnancy and hypertensive pregnancy. This may be the case of iNOS, which may have an important role in normal adaptive alterations of pregnancy [15] and contribute to vascular pathogenesis in pre-eclampsia, as suggested by the present findings.

To our knowledge, the effects of 1400 W on foetal development are not known, even though this iNOS inhibitor has been used in a number or animal studies and in a few studies in humans [38, 39]. Moreover, while there is evidence supporting a pathogenic role for increased oxidative stress in clinical pre-eclampsia [8, 40], most clinical trials do not support antioxidant therapy for prevention or therapy of pre-eclampsia [41, 42]. Therefore, caution is necessary to extrapolate our findings to the clinical setting. However, it is interesting to note that induction of haem oxygenase-1 decreased oxidative stress in the ischaemic placenta in the same RUPP model of pre-eclampsia used in this study, and this effect was associated with significant attenuation of hypertension [43].

In conclusion, our findings clearly implicate iNOS in the hypertension associated with experimental pre-eclampsia. It is possible that iNOS inhibitors may be clinically useful in the therapy of pre-eclampsia, especially in particular groups of patients carrying specific *iNOS* gene variants associated with increased iNOS expression or activity, which have been found more frequently in patients with pre-eclampsia [37]. Further studies in patients are warranted to examine this possibility.
